# ‘*I am the master key that opens and locks*’: Presentation and application of a conceptual framework for women's and girls' empowerment in reproductive health

**DOI:** 10.1016/j.socscimed.2020.113086

**Published:** 2020-08

**Authors:** Celia Karp, Shannon N. Wood, Hadiza Galadanci, Simon Peter Sebina Kibira, Fredrick Makumbi, Elizabeth Omoluabi, Solomon Shiferaw, Assefa Seme, Amy Tsui, Caroline Moreau

**Affiliations:** a Department of Population, Family and Reproductive Health, Johns Hopkins Bloomberg School of Public Health, USA; b Center for Advanced Medical Research and Training, Bayero University Kano, Nigeria; c Department of Community Health and Behavioral Sciences, School of Public Health, Makerere University, Uganda; d Department of Epidemiology and Biostatistics, School of Public Health, Makerere University, Uganda; e Centre for Research Evaluation Resources and Development, Nigeria; f Department of Reproductive Health and Health Service Management, School of Public Health, Addis Ababa University, Ethiopia; g Soins et Santé Primaire, CESP Centre for Research in Epidemiology and Population Health U1018, Inserm, F-94805, Villejuif, France

**Keywords:** Sub-Saharan Africa, Empowerment, Reproductive health, Fertility, Family planning, Qualitative research, Community norms, Women's health

## Abstract

*Rationale:* A renewed focus on women's and girls' empowerment in the era of Sustainable Development Goals reflects the belief that empowerment is central to health and development. Sexual and reproductive health (SRH) outcomes, including pregnancy and contraceptive use, may contribute to and result from empowerment. However, enhanced understanding of how women become empowered for SRH requires clear conceptualization of empowerment. *Objective:* We aimed to assess the applicability of a proposed framework for women's and girls' SRH empowerment (WGE-SRH) in sub-Saharan Africa. We sought to understand what shapes and motivates preferences for childbearing and contraception, exploring how women and girls navigate external pressures or rewards to exercise and achieve their reproductive goals. *Methods:* Grounded in the WGE-SRH framework, we conducted a qualitative study in four distinct contexts (Ethiopia, Kano and Anambra States in Nigeria, and Uganda). We implemented and analyzed 120 in-depth interviews and 38 focus group discussions with 440 women and men and translated results to refine the WGE-SRH framework. *Results:* Findings demonstrate the salience of women's internal motivations, including the social value and joys of motherhood, in shaping *existence of reproductive choices*. However, *existence of choice* was limited by couples' economic situations, pressures from providers, partners, and family members, and women's fears of contraceptive side effects or relationship dissolution. Despite these constraints, many found ways to *exercise their reproductive choices* through negotiation with partners, third party involvement in reproductive discussions, non-verbal communication, and covert use of contraception. *Conclusions:* The WGE-SRH framework is useful for exploring SRH empowerment, embracing the multilevel, dynamic nature of empowerment, as a process transitioning from *existence of choice* (autonomy) to *exercise of choice* (self-efficacy, decision-making, negotiation), and, ultimately, to *achievement of choice*. Future research and programs related to SRH empowerment should distinguish between *existence* and *exercise of choices* to promote the health and well-being of women and girls.

## Highlights

• First cross-country study of women's and girls' sexual and reproductive health empowerment (WGE-SRH) in sub-Saharan Africa.
• Internal and external factors, including economic contexts, pressures, and fear of consequences, shaped *existence of choice*.
• Women *exercise choices* by negotiating, involving others, communicating non-verbally, and using contraception covertly.
• *Existence* and *exercise of choices* are distinct elements of WGE-SRH that must be uniquely measured and addressed.
• Findings highlight the WGE-SRH framework's utility for monitoring SRH empowerment and outcomes in research and programs.

## 1 Introduction

### 1.1 A focus on empowerment in sexual and reproductive health

Family planning is a critical component of sexual and reproductive health (SRH), providing women with a means to prevent unintended pregnancies and achieve their reproductive goals. While the commitment to provide universal access to family planning services is reflected in Sustainable Development Goal (SDG) 3, barriers to satisfy women's demand for contraception remain pervasive due to misinformation about methods (Sedgh and Hussain, 2014; Sedlander et al., 2018), social stigma (Williamson et al., 2009), gender norms and religious beliefs about contraception (Sundararajan et al., 2019), economic constraints (Atake and Gnakou Ali, 2019), and lack of access to affordable and quality care (Williamson et al., 2009; Wood and Jewkes, 2006). A number of investments have sought to increase awareness of family planning and improve access to services, including media campaigns and strategies to reduce costs of commodities and services, enhance training of providers, and improve task-shifting of contraceptive services to lower level health workers (Mwaikambo et al., 2011). While utilization of family planning services has increased significantly since the launch of the SDGs, unmet need for contraception remains high, particularly in low- and middle-income countries (LMICs), thereby directing attention toward more distal, structural factors hindering women's SRH and well-being.

Acknowledging the constraints that unequal gender power dynamics have on women's lives, there is growing interest in promoting gender equality and women's and girls' empowerment (WGE) as a means to accelerate development and improve women's health and well-being (Kavanaugh and Anderson, 2013; Sonfield et al., 2013). A woman's ability to make strategic SRH decisions and act on those choices relates to her well-being, as evidenced by studies linking women's empowerment to improved SRH behaviors and outcomes (Prata et al., 2017), including use of contraception (Bose and Heymann, 2019; Ewerling et al., 2017; James-Hawkins et al., 2016; Yaya et al., 2018), prevention of unintended pregnancy (Upadhyay and Hindin, 2005), receipt of antenatal care (Mistry et al., 2009), improved maternal dietary practices (Gram et al., 2019), delivery with a skilled birth attendant (Shimamoto and Gipson, 2015), and negotiation in sexual relationships (Pearson, 2006; Wolff et al., 2000). However, a recent review on empowerment and family planning by Prata and colleagues reported inconsistent results regarding the contribution of empowerment to women's current use of family planning, which likely reflects differences in the conceptualization of empowerment across studies, outcomes examined in analyses, and normative views about family planning behaviors across populations (Prata et al., 2017). The spectrum of empowerment measures across settings, ranging from broad definitions of educational attainment, household decision-making, and mobility, which are primarily used in LMICs, to more specific indicators of SRH decision-making, generally used in higher income settings, expose the lack of a unified framework guiding research in this area.

### 1.2 Framing empowerment in sexual and reproductive health

Recent efforts to conceptualize empowerment generally, and SRH empowerment specifically, have contributed significantly to theoretical advancements (Bill & Melinda Gates Foundation, 2017; Donald et al., 2017; Edmeades et al., 2018; Ewerling et al., 2017; Malhotra et al., 2002). Extending Kabeer's foundational work on women's empowerment in the early 2000s, the World Bank developed a framework that defines empowerment as the interplay between resources and agency, which informs the progression from *existence of choice* through *exercise of choice* to *achievement of choice* (Kabeer, 1999; Malhotra et al., 2002). While the World Bank's framework elucidates the general process of empowerment, it does not specify the ways in which the model of empowerment pertains to specific life arenas, including sexual and reproductive decisions, actions, and outcomes. Several institutions, including the Royal Tropical Medicine Institute (KIT), in collaboration with the Bill & Melinda Gates Foundation (KIT-BMGF) (Bill & Melinda Gates Foundation, 2017), and the International Center for Research on Women (ICRW) (Edmeades et al., 2018), recently proposed two empowerment frameworks with a concerted focus on SRH. In addition to recognizing *choice* as a critical component of empowerment, the KIT-BMGF and ICRW frameworks explicitly acknowledge dimensions of *voice* and *power* as requisites for women to achieve their goals by challenging gender inequalities. Building on KIT-BMGF's model, the ICRW framework emphasizes SRH decision-making for critical behavioral domains (personal sexual relationships, reproductive control, and life choices); leadership in SRH (leadership roles in communal decision-making processes around reproductive health); and SRH collective action (influence over policies/programming and advocacy efforts). In contrast to the World Bank model, both the KIT-BMGF and ICRW frameworks draw less attention to the psychosocial processes guiding choices at the individual level, and instead, center more on programmatic and advocacy goals necessary to advance gender equality and women's empowerment at the societal level.

Despite these variations, all models share several unifying components that shape our understanding of SRH empowerment. First, empowerment represents both a process for achieving specific development outcomes, as well as a goal in and of itself. Second, empowerment is defined by the transition from one state to another; this transition involves the enhancement of one's ability to act on one's preferences. Third, empowerment involves individual attributes of agency, or an individual's ability to set goals and act on them. Fourth, external resources and opportunity structures, including access to education, employment, and financial resources, play a fundamental role in one's pursuit of her goals. Finally, all models identify power relations as a key obstacle to women's and girls' achievement of their goals. The World Bank's framework focuses on the internalization of these power structures at the individual level in an effort to describe how they contribute to individual goal-setting and actions, while the ICRW and BMGF models refer to power as a structural element that is situated outside of individuals. While we recognize the importance of structures and institutions in shaping women's choices and behaviors, and in advancing the empowerment of women and girls at the societal level, in this study, we focus on psychosocial processes and pathways linking choices to woman-centered outcomes at the individual level. Such pathways are more explicitly articulated in the World Bank's overarching framework, which informed our decision to test and adapt this framework to the study of SRH empowerment.

The need to ground SRH empowerment research in a theoretical framework, guiding the development of measures and the interpretation of results, is made more critical when comparing SRH empowerment across diverse populations. To date, studies on women's SRH empowerment have predominantly been conducted in Western Societies or South Asia (Prata et al., 2017), thereby limiting applicability to other contexts, including sub-Saharan Africa. In addition, a number of studies exploring SRH empowerment reflect programmatic or development goals, such as use of modern contraception, in lieu of women's and girls' own definitions of their SRH preferences, which vary according to their personal beliefs, values, and context (e.g., deciding if and when to have sex, use contraception, and bear children). While other studies consider some of these cultural specificities at the analytic stage (Malhotra et al., 2002), we sought to address these complexities at the conceptual and measurement stages by adapting our SRH empowerment framework to reflect common themes captured across socially and culturally diverse populations in sub-Saharan Africa. Although our approach focuses on commonalities, we recognize that intra- and inter-site variations in systems, including cultural and religious beliefs, health and disease burden, and governmental and societal structures, may not be thoroughly reflected. We believe this cross-cultural approach may serve as a better guide for developing SRH empowerment measures that are relevant across societies, and allow for comparisons over time and place to monitor progress toward development goals.

## 2 Objectives

This study aimed to assess the applicability of our proposed SRH empowerment framework and adapt the framework through the exploration of women's and girls' SRH empowerment across four geo-culturally diverse settings in sub-Saharan Africa. Our qualitative data collection and thematic analysis was guided by two research questions. First, we sought to understand what shapes and motivates women's preferences for childbearing and use of family planning. Second, we aimed to explore how women navigate external pressures or rewards to exercise and achieve their reproductive preferences.

## 3 A proposed conceptual framework for SRH empowerment

**Fig. 1:**
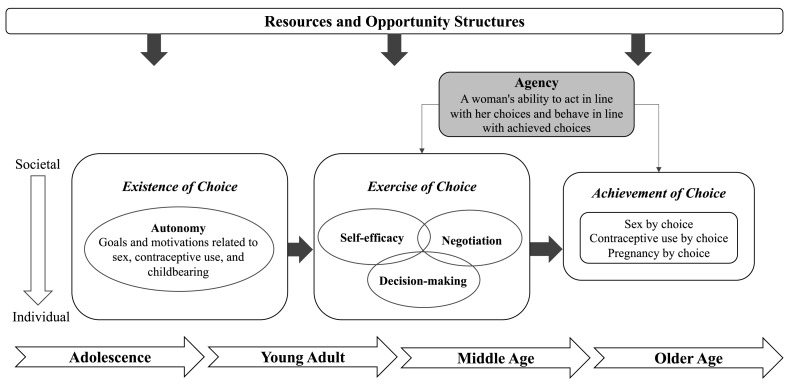
Women's and girls' empowerment in sexual and reproductive health (WGE-SRH) framework.

In this paper we briefly describe the process we used to develop, test, and refine an empowerment framework specific to women's and girls' individual SRH behaviors. We grounded the WGE-SRH framework in the World Bank's overarching empowerment framework and considered SRH empowerment as involving the progression from the *existence of choice* through *exercise of choice* to *achievement of choice* (Fig. 1). Our framework recognizes the power relations that operate at multiple levels, starting at the couple level, and expanding to the family, community, and societal levels, which collectively inform the ways individuals set and act on their SRH goals. Further, the framework acknowledges the important role of women's and girls' resources and opportunity structures, through education, economic conditions, and employment, in shaping women's and girls' SRH choices, actions, and achievement of reproductive goals. In addition, we recognize that the SRH empowerment of women and girls may change throughout the life course, following major life events, and in response to the intrinsic and extrinsic motivations that evolve during transitions from adolescence to middle and late adulthood (Lee-Rife, 2010).

Our woman-centered approach to framework development examines three specific SRH behaviors, defined as sex by choice, contraceptive use by choice, and pregnancy by choice. These outcomes reflect pertinent issues in women's and girls' lives, rather than those set by programmatic goals. A more detailed description of each dimension of the framework for pregnancy and contraceptive outcomes are the foci of this paper; the application of the framework to sex by choice has been described elsewhere (Wood et al., 2020).

*Existence of choice* corresponds to a woman's capacity to define her own reproductive goals. Our model was further informed by Donald et al.’s work, relating *existence of choice* to motivations or external pressures or rewards that women internalize to direct goal-setting. External motivations reflect both power relations with partners and family members or broader community norms valuing women's fertility (Donald et al., 2016). Beyond social and interpersonal motivations, women may also orient decisions within personal values and circumstances, such as their desires to become mothers, their educational or professional aspirations, or their financial circumstances (Bornstein et al., 2020). Our measures of *existence of choice* account for these varying, and sometimes conflicting, factors that shape women's and girls' goals related to pregnancy and contraceptive use.

Distinct from *existence of choice*, the second stage of the WGE-SRH framework, *exercise of choice*, examines the ability of women and girls to act on their SRH preferences. This step involves the activation of one's self-efficacy, decision-making abilities, and negotiation skills. Specifically, self-efficacy is an individual attribute representing a woman's confidence in voicing or acting on her reproductive preferences (Bandura, 1977), for example, a woman's confidence in her ability to avoid an unwanted pregnancy or use family planning. An individual's involvement in the decision-making process is a distinct construct informing her ability to act. Finally, negotiation skills reflect relational power dynamics that are particularly salient for pregnancy and contraceptive use, as they are behaviors that occur within a couple.

*Achievement of choice* is the final step of the empowerment process. In this framework, our reproductive outcomes are defined as: pregnancy by choice and contraceptive use by choice. By defining our outcomes as woman-centered, we sought to explore the influence of external factors across cultures and contexts through women's internalization of larger forces and pressures (e.g., community norms on individual's beliefs about the appropriateness of contraception for unmarried women). These outcomes were defined a priori by study teams given relevance across contexts and the potential for long-term monitoring within the broader study's platform.

## 4 Method

This multi-country qualitative study was conducted as part of the Performance Monitoring for Action 2020 (PMA2020) project designed to track progress towards the 2012 London Summit goal of providing family planning to an additional 120 million women globally by the year 2020 (Zimmerman et al., 2017). Data were collected between July and August 2017 to test and refine the WGE-SRH conceptual framework. A total of 120 in-depth interviews (IDIs) and 38 focus group discussions (FGDs; n=320 participants) were conducted with women aged 15–49 and men aged 18 and older across four study sites in three sub-Saharan African countries. A common cross-country research protocol was developed with local partners and implemented in each site. Institutional Review Board (IRB) approval was obtained from Johns Hopkins Bloomberg School of Public Health, Addis Ababa University (Ethiopia), Makerere University (Uganda), and the Ministries of Health of Anambra and Kano State (Nigeria).

### 4.1 Study context

Study sites included: 1) the Amhara region of Ethiopia, 2) Anambra state and 3) Kano state of Nigeria, and 4) select areas of Central/Eastern regions of Uganda. Sites were identified based on long-standing research collaborations and to represent a range of East and West African cultures at different stages of fertility transitions. Total fertility rates (TFR) in Nigeria and Uganda remain high at 5.3 and 5.4, respectively (2016 Uganda Bureau of Statistics (UBOS) and ICF, 2017; National Population Commission (NPC) [Nigeria] and ICF, 2018), with slow declines in recent years, while Ethiopia is experiencing a steady fertility decline, evidenced by a decrease in TFR from 5.4 in 2005 to 4.2 in 2016 (Central Statistical Agency and ICF, 2016). These four sites also exemplify diversity of structures applicable to understanding women's and girls' SRH empowerment. For example, Uganda's history of heightened HIV prevalence and Ethiopia and Nigeria's strong support for family planning programs characterize larger social, political, and cultural factors that affect SRH empowerment. While this study acknowledges the role of these factors, we focus specifically on cross-cultural commonalities in individual's psychosocial processes within these larger structures.

Given the significant geographical, religious, and cultural differences impacting SRH behaviors between northern and southern Nigeria, including differences in polygynous family structures and use of family planning services, Kano and Anambra states were each treated as independent sites for this study. These cultural variations are particularly important for the exploration of women's and girls' SRH empowerment across contexts. To identify commonalities, as well as context-specific attributes of women's SRH goals, decisions, and outcomes, according to their social and cultural environments, we conducted FGDs and IDIs in urban and rural areas in each of the four sites (Ethiopia, Anambra State, Kano State, and Uganda).

### 4.2 Qualitative instrument development and training

We developed semi-structured interview guides to align with the WGE-SRH conceptual framework, in order to explore sex, contraceptive use, and pregnancy by choice. The IDI guides probed into the personal experiences, perspectives, and narratives of women, girls, and their male partners related to decisions about if and when to engage in or abstain from sex, use contraception, and have children. The FGD guides focused exclusively on community beliefsabout these topics, from the perspective of rural and urban men and women at different stages of their reproductive lives. Comprehensive training, including an overview of qualitative research methods, in-depth review of the guides, and opportunities to practice and refine interviewing skills through mock interviews and pilot-testing, was conducted in each site. Guides were translated into and implemented in five languages: Luganda and Lusoga (Uganda), Amharic (Ethiopia), Igbo (Anambra, Nigeria), and Hausa (Kano, Nigeria).

### 4.3 Sample recruitment

**Fig. 2:**
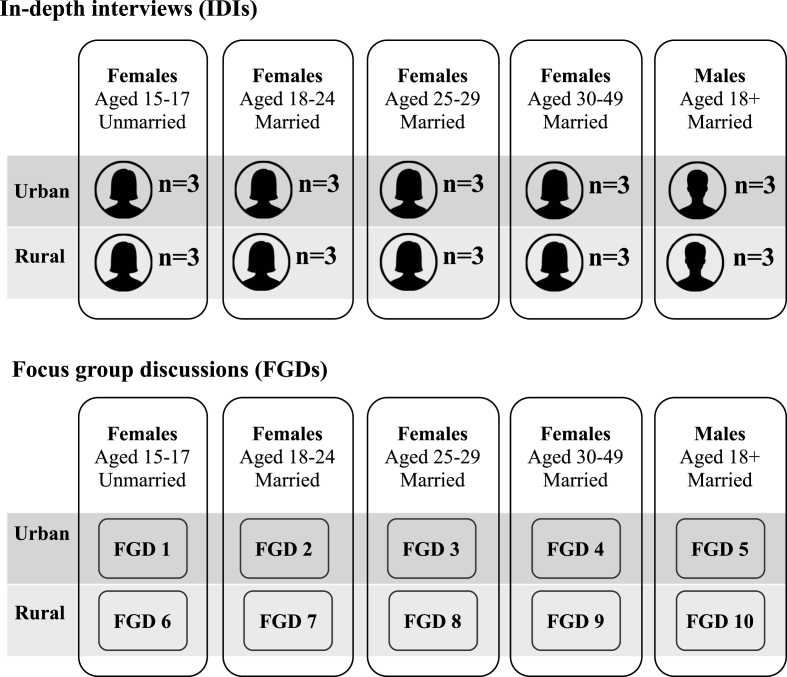
Composition of in-depth interviews and focus group discussions per site.

In each site, participants were recruited from one urban and one rural community. Prior to data collection, the in-country teams visited the study sites to meet with gatekeepers, community health teams, and local organizations, and confirm support referrals for women who reported experiences of intimate partner violence. Purposive sampling (by age, marital status, and area of residence) was used within each site to recruit women and men for IDIs and FGDs and to capture a diversity of motivations and actions underlying reproductive behaviors and outcomes (Fig. 2). Women aged 15–49 years (and men aged 18 or older whose wife was aged 15–49) who resided within the study area were eligible to participate. All women and men who were interested provided consent following IRB-approved standard ethical procedures.

### 4.4 Data collection procedures

FGDs were conducted in a private setting at a community facility, while IDIs took place in a private setting of the participant's choice or at the local study team's offices. Two team members were present during data collection; one served as the moderator and one as the notetaker. Each IDI/FGD lasted approximately 60–90 minutes. All discussions and interviews were digitally recorded with the permission of each participant. Upon conclusion of each session, the research team individually and privately administered a universal upset screener and provided participants with a list of local resources should they require additional support services.

### 4.5 Focus group discussions

Ten sex- and age-specific FGDs were conducted in each site, except Anambra where eight FGDs comprised of 10 people were completed (Fig. 2). Each FGD consisted of up to eight eligible, consented women or men (38 FGDs; n=320 total participants across four sites). Female and male FGDs were not linked and eligibility criteria did not necessitate partner inclusion. The number of FGDs reflects our interest in capturing the complexity of women's motivations to become pregnant or avoid pregnancy, and use or not use contraception, according to their social context (proxied by site and area of residence urban/rural)and their reproductive and marital experiences (unmarried versus married, nulliparous versus parous). FGDs were organized to allow maximum comfort and participation by grouping participants by site, sex, age, and marital status.

### 4.6 In-depth interviews

The majority of IDIs (n=96) were conducted with individual women and men from couple pairs. The remaining IDIs (n=24) were conducted with unmarried women across three age categories (15–17, 18–24, 25–49 years); inclusion of unmarried women was important to assess SRH empowerment outside of marriage (Fig. 2). Given the potentially sensitive nature of interviewing couple dyads, consent to participate in the IDI was first obtained from the woman. Following her interview, the woman decided if she permitted study staff to approach her husband for participation. This process sought to minimize risk of negative partner reactions, though no female partner declined to have her partner interviewed. IDIs were stratified by area of residence (urban/rural) and female age group to obtain diverse representation of participants.

### 4.7 Coding and analysis

Upon completion of data collection, in-country teams transcribed and translated audio files of into English with regular quality checks. Preliminary themes emerging from the data were shared across study sites during weekly calls. Emergent themes informed the development of a cross-site codebook; this codebook was inductive, using themes that emerged from the transcripts themselves, but was organized deductively to allow mapping of the codes to the WGE-SRH framework. Given cross-site differences, we also created site-specific codes for themes that arose exclusively in one site.

Following codebook development, in-country coding teams coded all transcripts. We used an incremental, step-wise approach to ensure quality coding; after every 10 transcripts were coded, two team members reviewed the coded quotes and discussed any discrepancies with the study team. We developed matrices, organized by codes, and mapped them to each dimension of the framework (*existence of choice*, *exercise of choice*, and *achievement of choice*) across the two outcomes (pregnancy and contraceptive use). Quotes from mapped codes were then categorized and organized by theme, sub-theme, and site for cross-site analyses. All analyses were conducted in Atlas.ti 8.

### 4.8 Framework revision

After cross-site data collection and analysis, we revised the framework according to the qualitative data. This iterative process of framework revision, with cross-cultural input, bolstered the framework's applicability across study sites. Specifically, the *exercise of choice* dimension was expanded from self-efficacy alone to further encompass negotiation and decision-making processes.

## 5 Results

Major themes and sub-themes are presented by domains of the WGE-SRH conceptual framework, with findings for *existence of choice* presented first. Given that the outcomes of pregnancy and contraceptive use are largely interrelated, results for the two outcomes are presented together.

### 5.1 Existence of choice for pregnancy and contraception

Women's *existence of choice* in decisions related to pregnancy and contraceptionwere deeply intertwined. These choices balanced personal maternal aspirations with expectations of partners, family, and communities regarding women's reproductive roles to advance social or family plans. Women's *existence of reproductive choices* changed across the lifespan. This dimension of empowerment was characterized by sanctions regarding premarital sexuality that limited the autonomy of unmarried women to use contraception or become pregnant, pressures to conceive early in marriage and “prove” one's fertility, and obligations governing females as responsible for childbearing decisions and practices later in marriage. *Existence of choice* for contraception also related to prior contraceptive experiences and interactions with providers, which informed women's current contraceptive preferences.

In the following section, we first discuss women's internal motivations for motherhood, as well as their perspectives on the social and familial benefits of controlling their fertility. Next, we discuss how gender expectations are reflected in the social pressures shaping women's childbearing and family planning decisions and how these expectations evolve over the life course. Finally, we discuss women's contraceptive preferences based on their experiences and misconceptions.

#### 5.1.1 Internal motivations to bear children and control one's own fertility

Women described their internal motivations to bear children, including achieving happiness associated with motherhood and gaining respect and social status. Children were discussed primarily as bringing joy to a couple and respect to the woman. Some women explained that bearing children was fundamental to a meaningful life, as participants in Ethiopia described:

[Children] are charming; you laugh when they laugh, and you feel sad when they become sad, which is a very good thing. Thus, it's good to have a child because their happiness makes you happy too.

-Female IDI participant, Rural Ethiopia, Age 22, Married

While motherhood was highly valued across settings, participants also discussed the benefits of regulating their fertility for personal advancement, such as to achieve educational and vocational aspirations. Many women reflected back on their reproductive lives, emphasizing the importance of establishing oneself with an education and career before marrying or giving birth. As participants from Ethiopia and Uganda described:

I did not think about getting pregnant and being a mother before marriage, but I thought about getting married after I completed my education and helping my parents… When I thought and discussed with my friends, my plan was always to get married after I completed my education and had my own job. As to my wish, I got married after my dream was successful.

-Female IDI participant, Urban Ethiopia, Age 25–29, Married

The conversation began when we stopped using condoms, and I was not willing to get pregnant because my family was the one paying for my tuition. I didn't want to disappoint them, so when we started engaging ourselves in such activities, I had to stand up for myself. I said, ‘You know what? We need to do this as a way to protect ourselves,’ so that is how we agreed.

-Female IDI participant, Urban Uganda, Age 25–29, Married

Women often described using family planning early in relationships as a mechanism to test their partners before committing fully through marriage or childbearing. Contraception was seen as an invaluable resource for protecting oneself from unintended pregnancy, particularly during the start of a new relationship before trust was developed. Family planning allowed women to determine if their relationships were strong, and if their partners were committed and able to provide for their family, before acting on their long-term reproductive goals.

As women advanced in their reproductive lives, family well-being was a major consideration in women's decisions to bear more children and was generally discussed in relation to economic circumstances. Financial status represented the largest constraint on women's *existence of choice* for pregnancy across sites. With the exception of northern Nigeria, large families no longer equated with high social status, but instead were considered in the context of the couple's economic capacity. Multiple men and women recognized the cost of raising additional children and the necessity of regulating their fertility to ensure a promising future for their children. In line with these economic considerations, participants discussed the changing social expectations surrounding childbearing in their communities, valuing quality of children over quantity.

I will never allow my neighbor [to] give birth without spacing. I might say [to] her, ‘It is enough as per your life, as per your economy.’ I may comment like this, but nowadays the community is on the way of modernization. From rural to urban, the communities give birth based upon their economy. Nowadays, I observe: while the rich are using family planning, controlling births and limiting their family, the poor families give birth without spacing. Even if I did not know their reasons, those families who had income wanted to limit their family.

-Female FGD participant, Urban Ethiopia, Age 30–49, Married

These economic considerations, however, were less prevalent in northern Nigeria, where personal and social motivations to bear more children were more common. In this context, where polygyny is a common family structure, men and women discussed women's motivations to secure their family positions of influence and inheritance through childbearing. In this context, competition between wives was a strong incentive to bear more children and reject family planning, even when husbands supported birth spacing.

Yes, the women in polygamy don't want to use family planning. They just want to give birth to as many children as possible. You will see some of the women having up to 30 children. The woman will say, ‘Why would [I] use family planning when the other wife is not using it?’ So, the wives will just be competing among themselves to give birth to as many children as possible.

-Female FGD participant, Urban Nigeria (Kano), Age 25–49, Married

#### 5.1.2 Gender expectations regarding childbearing, fertility control, and family planning

External pressures by partners, families, and communities over childbearing decisions were prevalent, reflecting the division of gender roles and power between male “producers” and female “reproducers” across sites. While premarital childbearing was negatively sanctioned, women were expected to “prove” their fertility as soon as they married by producing children for the family. In discussing premarital childbearing, participants emphasized the burden of an unintended pregnancy on women's social status, contrasting this with the positive experience of pregnancy among women who are married. As a young female participant in Nigeria noted:

It would be a pleasant experience to become a mother. If you are a mother, when you go out and when you return, your children will be running out to welcome you. However, becoming a mother is pleasant when you are legally married. If you have children, you will enjoy them. But if you are not properly married or have an accidental birth, you will not be proud of your child. You will be hiding the child.

-Female IDI participant, Rural Nigeria (Anambra), Age 16, Unmarried

After marriage, the social pressures reversed, emphasizing women's roles in the family as reproducers. Married women without children were disrespected, mocked, and seen as unproductive and hopeless; their partners were pressured to seek other partners who would bear children.

If I don't give birth after several years, people will definitely reduce the respect they give me. It is even possible for my relatives to start talking about me behind [my back]. They may start pressurizing me to marry another wife, or my wife's relatives may also want her to demand a divorce. So, these things can reduce the value and respect that someone has.

-Male IDI participant, Rural Nigeria (Kano), Age 18+, Married

In Uganda and northern Nigeria, participants described the childless women as “only eating the food and filling the toilet” to emphasize their perceived wastefulness in society. This view was less prevalent in Ethiopia, where both men and women discussed adoption as a possibility for couples who were unable to conceive children. FGD participants in Uganda detailed community perceptions about childless women:

R06: They are not viewed as women equal to those who can conceive. They say that they were simply brought to ‘fill the toilets’ and eat for nothing. [They] never have peace of mind, to the extent that children in the community undermine them and mock them.

R01: The woman without a child is often insulted and cannot say a thing about any other person's child. Even at the borehole when she tries to ask for water, she is insulted.

-Female FGD participants, Rural Uganda, Ages 25–29, Married

Within marriage, relational pressures, including fear of conflict, marital discord, dissolution and competition, also shaped women's *existence of choice* for contraception according to their partners' fertility desires. While some women discussed their childbearing intentions with their partners, others feared their partners' responses or abandonment if they did not accommodate.

It could be caused by men, whereby the woman is avoiding domestic violence. A man can tell a woman that, ‘If you do not want to produce for me children, I will get another woman’, so the woman will be put on pressure to produce many children to please the man and keep her marriage.

-Male FGD participant, Urban Uganda, Age 18+, Married

The choice is to not have a child. I will say, ‘This is my capacity and I will not give birth from now on.’ And then, if he says, ‘It is a must’, and I don't have capacity, there is nothing I can do. So, I will go and use contraception. After that, if he divorces me, he will do what he needs to do [seek others]. I mean, what can be done if he is arrogant?

-Female FGD participant, Rural Ethiopia, Age 25, Married

While men exerted a high level of control over childbearing decisions, women were compelled to translate these intentions into action. Thus, women were expected to take full responsibility to control their childbearing practices, including understanding, monitoring, and controlling their fertility, in accordance with their partner's reproductive goals. As a result, women were blamed for failing to control their fertility, and women who had children “back-to-back” were disrespected in the community and seen as irresponsible.

What I am saying is, as a man, what you know is just to meet with your wife. She is the one that knows when she is ripe to conceive or not. So, if you are doing everything to make her get pregnant, she is the one that knows when you will meet her. She will become pregnant and she knows what to do not to become pregnant, even if you do all you want to do.

-Male FGD participant, Urban Nigeria (Anambra), Age 35, Married

A person with many children is always worrying because people will say, ‘That [woman] gave birth to so many children!’ The neighbors will get stressed when their colleagues say, ‘It's too unfortunate for [that woman] to have all those children.’ So, in most cases these children became a burden to the neighbors by coming around to wait for food. People will say you gave birth to so many children, yet you have nothing to feed them.

-Female FGD participant, Rural Uganda, Age 30–49, Married

In line with this gender division of reproductive roles, participants across sites identified family planning as a woman's matter, with limited partner knowledge or engagement in contraceptive decisions. These gender expectations were often characterized as women “knowing how their bodies work”, and being the ones who plan when to get pregnant, raise the children, and have more contraceptive method options than men. The gender gap in knowledge about contraception often contributed to women gaining effective control over their fertility. Even when their partners claimed the right to make childbearing decisions, women's knowledge of fertility regulation and family planning often enabled them to take sole responsibility for contraceptive decisions.

I think the other person who can decide that apart from God is the woman, because it is the woman who suffers when she gets pregnant. Women struggle a lot with the children so they decide for themselves

-Male FGD participant, Urban Uganda, Age 18+, Married

#### 5.1.3 Family planning preferences and health

While gendered division of roles over childbearing decisions governed women's motivations, their preferences were also conditioned by health concerns, previous experiences, and healthcare interactions. Health concerns were prevalent, particularly among multiparous women who discussed the value of contraception in averting harm and preventable deaths from pregnancies spaced too closely. As a young married woman in Kano explained:

When I got pregnant quickly after my first child, the second one [caused me to suffer], and I had complications because [my pregnancy] did not have proper care… [Family planning] is important to your personal health and that of your family at-large.

-Female IDI participant, Rural Nigeria (Kano), Age 18–24, Married

Personal experiences and community discussions related to positive and negative effects of modern contraceptive methods, including menstrual disturbances and weight change, were prominent drivers of women's decisions to use or abstain from modern contraception.

I only like the implant because I have used it before and it suits my body. Apart from that, I don't think I like any other one.

-*Female IDI participant, Urban Nigeria (Anambra), Age 27, Married*

Again, I might secretly go for maybe a coil or pills, but the challenge is getting [side effects]. I remember one time I secretly went for an injection and bled heavily for four months. This made me decide to tell him; though he quarreled, the issues were solved.

-Female *FGD participant, Rural Uganda, Age 30–49, Married*

Women in Uganda also emphasized the negative impact that contraception could have on their sexuality:

R07: It can extend your periods abnormally and also make you lose sexual urge and appetite for food.

R06: Family planning methods can cause vaginal dryness that when you have intercourse you end up sustaining wounds due to the lack of vaginal fluids.

-Female FGD participants, Rural Uganda, Age 25–29, Married

While some women reported side effects they experienced, misconceptions and perceptions of peers' side effects often drove women's resistance to using contraception, particularly for women in Ethiopia and Uganda. As one woman in Ethiopia explained, “Yes, my sister; since [family planning] brings uterus narrowness, you should not use it,” (Female IDI participant, Rural Ethiopia, Age 17, Unmarried).

Additionally, many women were concerned that contraception could cause infertility. Given the social value placed on one's fertility, using contraception before childbearing was perceived as risky, especially among young women. As women in Uganda noted, “They say that [family planning] makes one barren because it burns the ovaries,” (Female IDI participant, Urban Uganda, Age 18–24, Unmarried). Men also shared these concerns regarding infertility after family planning use.

In regards to the planning [family planning], at the moment if there is a [family planning method] that I know has no problem or effect, I can do it. But, based on the people I've seen who used it, I saw that they suffered a lot when it comes to getting pregnant. That is why it scares me.

-Female *IDI participant, Urban Nigeria (Kano), Age 25–49, Married*

For example, if a woman uses contraceptives and is unable to conceive due to natural reasons or is suffering from delayed fertility, but she says she couldn't have a child after using contraceptives, the society ultimately perceives that the infertility is because of contraceptives and won't use [family planning].

-*Male* FGD *participants, Rural Ethiopia, Age 18+, Married*

Some of these concerns were lifted by interactions with healthcare providers. Many women referred to healthcare providers, including doctors, community health workers, and nurses, as knowledgeable influencers of their reproductive decisions and recognized these individuals as providing reliable information to guide their method selection.

The health workers are blessed with the knowledge concerning health, the advantages and the disadvantages of all the methods. If I want to engage in [family] planning, I am supposed to contact them to help me make a choice through their knowledge.

-Female IDI participant, Rural Nigeria (Kano), Age 18–24, Unmarried

However, a few women also discussed coercive experiences with providers who denied them access to methods or pressured them to use certain methods, particularly in Ethiopia and Uganda. FGD participants from Uganda illustrated the various ways that healthcare providers restrained women's *existence of choice* and access to family planning methods, including forcing women to wait until they had more children, delaying until the next menstruation, or requesting payment before providing services:

R02: Some health workers discourage women depending on the number of children they have, even when the woman is in need [of family planning]. This affects the woman's peace of mind. [The health workers] also tell people that they cannot manage family planning.

R03: Some of them first ask you if you have [menstruated], and if not, they tell you to first go back and wait until [menstruation], then you return. Yet, your husband may not wait and within that period you can get pregnant.

R06: Some health workers tend to ask for money before administering the injection. This discourages the woman and affects their thoughts.

-Female FGD participants, Rural Uganda, Ages 25–29, Married

### 5.2 Exercise of choice for pregnancy and family planning

Despite these limitations on women's *existence of choice*, some women exercised their childbearing and family planning choices by using contraception covertly, seeking guidance from and involving third parties in discussions about family planning, and ultimately acting as the final decision-makers in order to achieve their reproductive goals.

#### 5.2.1 Covert use of contraception to achieve reproductive goals

In the face of opposition, many women found strategies to avert short pregnancy intervals with or without their partners' knowledge. To act on their reproductive goals, women relied on covert use of contraception. As a woman in Ethiopia said: “If I did not agree with my husband's suggestion, I can avoid pregnancy by using contraception—by hiding from him,” (Female IDI participant, Rural Ethiopia, Age 18, Married). Some women used contraception covertly due to known opposition of the partner, whereas others exercised their contraceptive choices on their own, without partner involvement in reproductive decisions.

Yes, she is the one suffering. He doesn't know what she is going through. His own [interest] is to see her pregnant and see the children in front of him. He doesn't want her to do family planning, so she will quietly go and do it. He will not know; [he will] only know that he will not see her pregnant.

-Female FGD participant, Urban Nigeria (Kano), Age 18+, Married

You just have to do things secretly. If you have decided to go for pills, then it has to be secretly, without the man's knowledge. If it's injections, you can go for it without getting him to know. He will keep asking why you are not conceiving, but when it's your secret.

-Female *FGD participant, Rural Uganda, Age 30–49, Married*

Many men emphasized their preference for joint decision-making and agreement, while often acknowledging their partners were using contraception covertly.

If they have both decided to use the contraception together, it has a big benefit. But if the woman decided on it on her own, she is the one who has benefited because he doesn't know anything. If one day he wants a child, and if she is using without his knowledge, he may not like it when he finds out later.

-Male FGD participant, Rural Ethiopia, Rural Male, Age 26, Married

Women also highlighted the marital issues that could result from partners’ discordant fertility intentions and related consequences of covert use of contraception. Responding to threats or experiences of reproductive coercion, some women would exercise their reproductive choices by counter-threatening divorce or separation.

What I mean, for example, if he forced me to give birth, I will say, ‘No, I will not live with you giving birth without my will; it is enough,’. But if he insists me to give birth, I will be divorced with him;I have no other choice. So, I don't need to give birth, I will leave him.

-Female FGD participant, Urban Ethiopia, Age 32, Married

#### 5.2.2 Role of friends, family and community members in support of women's exercise of choice

Participants across sites emphasized the role of friends, family, and community members in helping couples manage disputes about their reproductive goals. Women engaged these individuals in conversations with their partners as a strategy to exercise their choices when the couple disagreed about childbearing decisions. Third parties were largely involved to support the woman by persuading her husband to use family planning to space pregnancies; these conversations often centered on protecting the woman's health and ensuring financial stability to raise the children.

When I first told him, he refused. He said I should have two more [children] and add to the ones I already have, so that we will know we have finished giving birth. I told my mother-in-law; she said it is okay, that she will reason with him. So, she called him and told him. He now accepted what his mother told him. After that he called me, and we finalized and concluded on our decision not to have any more children until later.

-Female IDI participant, Urban Nigeria (Anambra), Age 24, Married

Members of social support networks, including, relatives, community members, and religious leaders, were instrumental in not only mediating partner conversations, but also in helping women obtain contraception covertly, if necessary. For example, a community education and outreach initiative led by the Ministry of Health in Ethiopia, called ‘1-to-5 groups’, was central to women's ability to navigate these situations:

If he didn't agree [with her] and said, ‘You have to give birth now’, or if she said, ‘I have to give birth now. I don't want to use a contraceptive [method],’ he would make her be advised by her relatives. He would make her be advised by friends, by her sister, and brother. He would make her advised saying, ‘We have to wait some time. We have to delay it, and we have to strengthen our life first.’ Whereas if she is willing and he didn't agree, she will discuss about it with 1-to-5 group leader. She will go with the 1-to-5 group leader and will start using by secret.

-Female FGD participant, Urban Ethiopia, Age 49, Divorced

#### 5.2.3 Women as the ultimate decision-makers for reproductive decisions

Participants highlighted women's ultimate decision-making power for family planning, whether it was in support of family planning or against it. For example, some participants described how they could actively avoid using family planning, even if their partners wanted to prevent pregnancy. In this regard, women discussed the control they exerted over these types of reproductive decisions in their partnerships. As participants in northern Nigeria and Uganda stated:

If it happens that her husband does not care about her health or if he does not care to support her, if she goes ahead with it, she is right. How can she keep suffering herself? Whenever she gets pregnant, she will keep managing with it and she is not being looked after. When she goes to obtain family planning, you won't say she is not right.

-Male FGD participant, Rural Nigeria (Kano), Age 18+, Married

I am the master key that opens and locks, so I decide what to do. If you tell me to use family planning, yet I am not interested, I can easily lie to you that I received an injectable and I can end up pregnant. So, as women, we make the final decision.

-Female FGD participant, Urban Uganda, Age 25–29, Married

## 6 Discussion

In this study, we applied the proposed WGE-SRH framework among women and men across four culturally diverse settings throughout sub-Saharan Africa to explore their perceived *existence* and *exercise of choices* about pregnancy and contraception (Fig. 1). The narratives shared by participants embed well within the framework, examining women's and girls' SRH empowerment throughout the life course. The present study extends prior research by pursuing a cross-cultural approach to incorporate the perspectives of women from different socio-cultural contexts who were at different stages of their reproductive lives to investigate SRH empowerment across sub-Saharan African contexts. The study also builds on a body of literature that is largely void of male perspectives, despite their involvement in SRH outcomes, by examining the views of male partners on women's decision-making and behavioral authority over SRH issues.

We found the WGE-SRH framework proved useful for exploring common cross-site themes, while also recognizing the importance of cultural specificity. By approaching the investigation of women's and girls' SRH empowerment, centering on individuals' psychosocial processes, we were able to disentangle internal and external motivations of women, their partners, families, and communities to understand SRH decisions and distill themes across contexts. The cross-site themes underscore the distinct roles that *existence* and *exercise of reproductive choice* play in shaping women's decisions and actions that lead to pregnancy and contraceptive use by choice. While previous studies have discussed components of *existence* and *exercise of choice*, no prior researchhas combined them into a unified framework to examine woman-centered SRH empowerment outcomes.

Our findings on women's *existence of reproductive choice* reinforce research conducted in sub-Saharan Africa, demonstrating similar constraints on women's autonomy for pregnancy and family planning decisions (Capurchande et al., 2017; Lodenstein et al., 2018; Maharaj and Cleland, 2008; Mosha et al., 2013; Upadhyay et al., 2014). Social expectations related to women's roles in relationships and reproduction, particularly that women are charged with bearing children soon after marriage, impeded young women's *existence of reproductive choices*. Prominent in women's *existence of reproductive choices* were their internal motivations, including desires to complete their education, raise children of quality, and protect their health.

However, widespread fear of consequences related to contraceptive use, often stemming from misconceptions or fears about methods and perceived side effects, hindered women's *existence of reproductive choices*. The internalization of these fears, including the belief that contraception caused infertility or that side effects could lead to relationship dissolution, directly conflicted with women's responsibility to control reproductive matters within their partnerships. These findings build on previous literature about community perceptions of infertility and the constraint that fear of infertility, manifested through contraceptive myths and misperceptions, have on women's contraceptive autonomy (Bornstein et al., 2020; Ochako et al., 2015; Sedgh and Hussain, 2014; Sedlander et al., 2018; Williamson et al., 2009; Wood and Jewkes, 2006). These social constraints were, to some extent, buffered by a number of strategies that enabled women to exercise their reproductive choices.

The *exercise of choice* findings highlight the complicated decisions women make when choosing whether or not to engage their partners in contraceptive decision-making, as well as the unique roles of family, friends, and community members in helping women pursue their reproductive goals. While these findings are consistent with qualitative research in similar settings (Castle et al., 1999; Heck et al., 2018), they also highlight limitations of current quantitative measures, which may not effectively capture the nuances of women's decisions to use contraception covertly and the potential ramifications related to being discovered (Choiriyyah and Becker, 2018, Kibira et al., 2020; Levinson et al., 1998). Confidence in a woman's ability to discuss family planning with her partner is a relevant measure of contraceptive self-efficacy (Levinson et al., 1998). However, such a measure may fail to comprehensively ascertain the *exercise of choice* dimension, given that many women are able to achieve their reproductive goals regardless of the verbal communication they engage in with their partners.

Our findings indicate the need to expand the *exercise of choice* domain to encompass a more holistic definition of acting on one's choices, characterizing it as unique from “voice” dimensions specified by other frameworks (Bill & Melinda Gates Foundation, 2017; Edmeades et al., 2018). This emphasis on distinguishing between the components of *exercise of choice* also reinforces recent findings that different factors influence communication and decision-making, which both contribute to reproductive autonomy (Loll et al., 2020). Future research to examine covert use of contraception should explore women's motivations, and the role of partners and third parties in couples' discussions and decision-making related to family planning, as well as potential repercussions of disclosing contraceptive use. Moreover, we urge researchers to develop and utilize more encompassing measures of *exercise of choice* that highlight these complex decision-making processes, beginning with women's confidence in initiating discussions about childbearing and contraception, extending through the negotiation of contraceptive use, and finally, disentangling decision-making roles and consequences of women's actions.

Echoing the findings of research on women's economic, educational, and vocational outcomes, our study found that gender expectations are a key structural system shaping women's reproductive decisions (Nanda et al., 2013; Ninsiima et al., 2018). By dividing gender roles and power, these systems limit women's and girls' control of their reproductive lives and hinder their ability to achieve their individual reproductive goals (Connell, 1987; Rosenbaum et al., 2016). Specifically, women were responsible for implementing all childbearing and family planning decisions, which largely reflected their partner's childbearing choices.

The only site in which women's roles related to childbearing diverged was Kano, where women in polygynous marriages reigned supreme over pregnancy decision-making within the couple. Both men and women discussed a lack of male autonomy in these decisions when multiple wives desired children. These results contrast with previous qualitative studies in two difference cultural settings, Tanzania and western Kenya, which highlight men as the ultimate decision-makers in reproductive matters (Mosha et al., 2013; Withers et al., 2015). While participants emphasized women as decision-makers, they also described the consequences that women faced if negative events occurred, for example an unintended pregnancy, a delay in becoming pregnant, and the experience of side effects from contraceptive use. These results underscore the complexity of women's responsibility for, practice of, and repercussions related to reproductive decision-making within a couple. Further, these findings enforce the importance of acknowledging and addressing the role of gender inequity in decision-making related to childbearing and family planning, both within couple dyads and larger societal structures.

### 6.1 Limitations

Our study is not without limitations. First, questions on sensitive topics related to pregnancy and family planning in FGDs may have constrained the range of discussions among women about the topics, thereby limiting the breadth of information we obtained. However, we strived to address this potential bias by composing FGDs in age- and marital-specific groups to maximize comfort and open dialogue (Fig. 2). Second, although couples were interviewed independently, their interviews were not linked, thereby limiting examination of SRH empowerment within couples. Despite this limitation, we investigated individuals' perspectives of how their partnerships influenced women's existence of choice and ability to exercise and achieve their reproductive goals.

Third, due to our specific focus on women's individual expressions of SRH empowerment, we did not explore women's ability to exercise collective agency, or group action. Likewise, we did not examine women's ability to gain resources, like economic independence and educational attainment, in order to exercise their SRH choices, or the influence of these contextual factors on women's and girls' SRH empowerment directly. Recognizing that women are situated within the contexts of their opportunity structures, we urge future research to explore how women mobilize resources and exercise SRH agency, while accounting for their broader circumstances (see Fig. 1).

Fourth, by narrowing in on commonalities across sites, we were not able to thoroughly acknowledge site-specific intricacies and histories, including structural factors specific to distinct communities and cultures, that reinforce or prevent women's *existence* and *exercise of choice* for SRH decisions. Finally, in FGDs, it was difficult to disentangle women's personal experiences using contraception from the misconceptions they held about specific methods. The interrelated nature of experienced side effects, perceived side effects learned from social networks, and the experiences of friends and family made it challenging to examine the different ways side effects and misconceptions constrained women's *existence of choice* for contraception.

### 6.2 Implications

Interventions and programs that aim to improve women's and girls' SRH empowerment should uniquely address the different factors at the societal, community, family, and couple levels constraining *existence* and *exercise of reproductive choices*. Accounting for the distinctions between these levels of influence on the empowerment process, programming to improve women's *existence of choice* for reproductive health decisions should focus on challenging gender norms and hierarchies within communities. Further, efforts to enhance women's and girls' *exercise of choice* entails supporting providers to deliver patient-centered and respectful family planning counseling to meet women's preferences and goals. At the couple level, male partner engagement in discussions and education surrounding reproductive health and family planning is crucial to ensuring that women are able to communicate their preferences and negotiate to exercise their choices. Finally, interventions should adopt woman-centered outcomes aligned with women's and girls' reproductive goals, instead of programmatic outcomes that may not translate directly into their preferences or needs. In recognizing that reproductive preferences vary between individuals, this approach can increase the ability of women and girls to achieve and sustain their reproductive goals.

The emergence of common themes that guide women's decisions and abilities to act on SRH preferences across very diverse social and cultural settings also provides an opportunity to develop quantitative measures, grounded in the WGE-SRH framework. Such development would enable researchers and practitioners to monitor SRH empowerment across time and place and evaluate intervention effectiveness. Since its development and refinement, the WGE-SRH framework has been translated into and pilot-tested as an index of women's and girls' SRH empowerment across the four study sites (Moreau et al., 2020).

## 7 Conclusion

This study comprehensively explores women's and girls' sexual and reproductive empowerment across four distinct contexts in sub-Saharan Africa. The qualitative findings support the WGE-SRH framework as a useful model for examining how societal, community, family, couple, and individual influences shape reproductive goals and the ability of women and girls to act on their preferences. Specifically, the WGE-SRH framework is central to understanding the psychosocial processes, from personal aspirations, to couple dynamics to community norms, shaping women's *existence* of reproductive choices and their ability to *exercise these choices* leading to achieved reproductive goals.

Women's and girls' empowerment for SRH remains central to global development goals, especially those related to increased gender equality and universal access to SRH services. The multilevel factors, highlighted in the WGE-SRH framework and throughout the qualitative interviews, simultaneously create opportunities and barriers for women in pursuit of their reproductive goals. Interventions and programs can be oriented within the WGE-SRH framework to target specific constraints and motivations and optimize the ability of women's and girls' to recognize and act on *existence* and *exercise of choices* to achieve their reproductive goals.

## Author credit statement

**Celia Karp**: Conceptualization, Methodology, Project administration, Data curation, Validation, Visualization, Formal analysis, Roles/Writing - original draft. **Shannon N. Wood**: Conceptualization, Methodology, Project administration, Data curation, Validation, Visualization, Formal analysis, Roles/Writing - original draft. **Hadiza Galadanci**: Conceptualization, Project administration, Supervision, Investigation, Writing - review & editing. **Simon Peter Sebina Kibira**: Conceptualization, Methodology, Project administration, Supervision, Investigation, Writing - review & editing. **Fredrick Makumbi**: Conceptualization, Project administration, Supervision, Investigation, Writing - review & editing. **Elizabeth Omoluabi**: Conceptualization, Methodology, Project administration, Supervision, Investigation, Writing - review & editing. **Solomon Shiferaw**: Conceptualization, Project administration, Supervision, Investigation, Writing - review & editing. **Assefa Seme**: Conceptualization, Project administration, Investigation, Supervision, Writing - review & editing. **Amy Tsui**: Conceptualization**,** Methodology, Supervision, Writing - review & editing, Funding acquisition. **Caroline Moreau**: Conceptualization, Methodology, Supervision, Formal analysis.
